# Exploring the mechanisms of mutual influence between lactylation and macrophage polarization in the context of disease

**DOI:** 10.1002/ctm2.70499

**Published:** 2025-11-05

**Authors:** Houhua Guo, Nannan Luan, Jian Gao, Xiaoao Pang, Jianlei Bi, Liancheng Zhu

**Affiliations:** ^1^ Department of Obstetrics and Gynecology Shengjing Hospital of China Medical University Shenyang Liaoning China; ^2^ Department of Obstetrics and Gynecology The Second Hospital of Dalian Medical University Dalian Liaoning China

**Keywords:** immunity, lactylation, macrophages, post‐translational modifications, tumours

## Abstract

**Background:**

Lactylation, a post‐translational alteration facilitated by lactic acidderived lactyl‐CoA, has emerged as an epigenetic regulator that alters gene expression in macrophages. Emerging data situates lactylation at the nexus of metabolic flux and immune cell destiny, especially in tumor and inflammatory microenvironments.

**Main text:**

Lactylation is significantly linked to tumor progression and the polarization of macrophages towards the M2 phenotype, a condition that exacerbates cancer and associated inflammation. Modulating lactylation levels can alter the M1/M2 balance, hence affecting the progression of cancer and inflammatory illnesses. These findings identify lactylation as aregulator that can either suppress or enhance tumor development and the related inflammatory response, contingent upon the context and degree of the change.

**Conclusion:**

This review systematically elucidates the role of lactylation in directing macrophage polarization in the context of cancer and associated inflammation. The aggregated data suggest that targeting lactylation constitutes an innovative therapeutic strategy for regulating immune cell activity and managing the advancement of cancer and related inflammatory conditions.

**Key points:**

- The conversion of lactate to lactyl‐CoA facilitates enzymatic histone lactylation, transforming glycolytic byproducts into an epigenetic regulatory mechanism for gene expression.
- Lactylation modification influences macrophage polarization towards M1 or M2 phenotypes, affecting outcomes in infection, cancer, and fibrosis.
- Targeting lactylation modifiers through pharmacological means introduces a novel metabolic‐epigenetic approach for treating disorders.

## Graphical abstract

1. The conversion of lactate to lactyl‐CoA facilitates enzymatic histone lactylation, transforming glycolytic by‐products into an epigenetic regulatory mechanism for gene expression.
2. Lactylation modification influences macrophage polarization towards M1 or M2 phenotypes, affecting outcomes in infection, cancer and fibrosis.
3. Targeting lactylation modifiers through pharmacological means introduces a novel metabolic‐epigenetic approach for treating disorders.

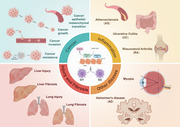

## 1 INTRODUCTION

Epigenetic alterations act as pivotal controllers of genetic expression, influencing inheritance patterns, disease progression and developmental processes through mechanisms such as DNA methylation, histone post‐translational modifications (PTMs), nucleosome remodelling and non‐coding RNA interactions.^1^ As the fundamental building blocks of chromatin, histones undergo a diverse array of PTMs^2^—including acetylation, ubiquitination and methylation—that involve the reversible attachment or removal of chemical moieties (e.g. methyl, acetyl or phosphate groups) on specific amino‐acid residues.^3^ These modifications alter the physicochemical properties of histones and endow precursor proteins with distinct biological activities, thereby fine‐tuning gene expression.

Recent advances in mass spectrometry have unveiled a novel histone PTMs, lactylation.^4^ Functionally, lactylation becomes particularly prominent during the late phase of M1 macrophage polarization; under steady‐state conditions, H3K18 lactylation directly drives the transcriptional activation of genes associated with the M2 phenotype.^4^ Importantly, alongside this newly identified lactylation, a spectrum of PTMs—including acetylation, phosphorylation and others^2^—plays pivotal roles in the pathogenesis of numerous diseases such as neurological disorders, metabolic dysregulation, cardiovascular disease, inflammatory states and cancer.^5^

Macrophages are vital immune effector cells that perform multiple functions during normal physiological balance and in response to immune challenges. Their functional plasticity stems from inherent diversity and adaptability. Upon polarization, macrophages adopt either pro‐inflammatory (M1) or anti‐inflammatory (M2) properties. M1 macrophages predominantly rely on glycolytic metabolism to initiate and sustain pro‐inflammatory responses, mechanisms linked to both tissue damage exacerbation and enhanced anti‐tumour immunity.^6^, ^7^ Conversely, M2 macrophages preferentially utilize oxidative phosphorylation pathways. This metabolic signature supports their roles in inflammation resolution, clearing parasites and paradoxical tumour promotion, while also concurrently facilitating tissue repair and immunomodulatory functions.^2^, ^3^ Disease pathogenesis frequently correlates with dynamic shifts in macrophage polarization states,^8^ highlighting the role of macrophage polarization control in immune system regulation.^9^

The interaction of lactylation and macrophage polarization phenomena constitutes pivotal regulatory mechanisms in cellular pathophysiology. These processes exert substantial influence on immunological homeostasis, inflammatory cascades and oncogenic progression. Exploring their mechanistic link enhances insights into disease development and uncovers new precision medicine targets. Emerging evidence suggests that pathological lactylation aberrations may drive disease progression through epigenetic modulation of macrophage polarization states, thus establishing a critical nexus for therapeutic strategies targeting immune‐metabolic reprogramming.

## 2 LACTYLATION AND MACROPHAGE POLARIZATION

### 2.1 The uncovering of lactylation

Previously, lactate was thought to be a by‐product of the body's metabolism, whereas glucose served as fuel.^10^ Recent studies have shown that lactic acid serves both as the final product of glycolysis and as a signalling molecule in hypoxic environments. Monocarboxylate transporters (MCTs) may move lactate across the membrane. MCT1 is in charge of bringing lactate in, and MCT4 is in charge of sending it out.^11^ The particular receptor GPR81 can also find high quantities of lactic acid that have built up outside of cells. It starts signalling pathways inside cells when it binds to GPR81.^12^ So, MCTs are mostly in charge of moving lactic acid around, whereas GPR81 is in charge of sensing the amount of lactic acid outside the cell and controlling its signalling function. According to recent research, lactate, besides being the conclusion of glycolysis, also facilitates signalling by crossing membranes. This signalling occurs through several MCTs and their receptor GPR81 under hypoxic conditions^12^ and to high extracellular lactate levels. The primary lactate transporters are MCT1, which imports lactate, and MCT4, which exports lactate.^11^ Beyond its metabolic role, lactate acts as a common energy source for various biological processes, performing both metabolic and non‐metabolic activities in pathological processes.^13^ Lactate thus functions dually as both an energy substrate and a pathophysiological regulation. Research has shown that it can drive the growth of tumours,^14^ dampen the activity of immune cells that have infiltrated the tumour microenvironment (TME)^15^ and contribute to inflammatory, cardiovascular and neurological pathologies.^15^, ^16^, ^17^

Additionally, lactate originating from glycolysis facilitates communication between cellular metabolism and epigenetic control through histone alterations.^18^ In 2019, Professor Zhao's research team made a major discovery. They revealed lactylation‐induced histone lysine lactylation‐modification (Kla), a new form of epigenetic regulation that affects the expression of genes involved in metabolism.^4^ Using integrated HPLC‐MS/MS analysis detecting characteristic 72.021 Da mass shifts (consistent with lactyl group adducts), anti‐Kla immunoblotting validation and SILAC‐based quantitative proteomics, they systematically demonstrated the broad conservation of this modification across biological systems.

### 2.2 Widespread lactylation modifications

Lactylation is a prevalent post‐translational alteration impacting histones and non‐histone proteins, establishing a bifunctional regulatory mechanism through distinct modification pathways. Histone lactylation exerts epigenetic control via three principal modalities: (1) chromatin structural remodelling, (2) modulation of transcription factor‐DNA interactions and (3) regulation of promoter region accessibility. Conversely, non‐histone lactylation mediates functional alterations through steric hindrance, conformational dynamics and charge redistribution, thereby governing enzymatic activity, protein–protein interactions and intracellular trafficking.^19^ Pioneering work by Moreno‐Yruela et al. revealed extensive lactylation patterns across human tissue proteomes, including glycolytic enzymes and nucleolin modifications at K102/K116 residues.^20^ Additionally, HMGB1, a widespread, non‐histone protein in eukaryotic cells, is also lactylated.^21^

Research into lactylation positions revealed 2375 Kla sites in 1014 proteins within gastric cancer cell samples.^22^ Wu et al. identified 2045 Kla‐modified sites across 960 proteins in hepatocellular carcinoma,^23^ while Yang et al. discovered 9256 Kla modification sites on non‐histones, indicating that lactylation extends beyond histones and transcriptional regulation.^24^ Lactylation is not exclusive to humans; it also occurs in other species. Zhang et al. pinpointed 387 lactylation sites across 257 proteins within the *Trypanosoma brucei* parasite, a causative agent of African sleeping sickness.^25^ Yin et al. documented an extensive analysis of *Toxoplasma gondii* (RH strain), uncovering 1964 Kla modification sites distributed across 955 distinct proteins.^26^ Meanwhile, Li's research team conducted a parallel investigation into *Frankliniella occidentalis*, identifying 1458 lactylation sites spanning 469 different proteins.^27^ Li et al. documented 1869 Kla sites within 469 proteins included in the carcinogenic strain of *Streptococcus mutans*.^28^ A total of 868 lactylation sites were mapped across 379 proteins in the *Nannochloropsis oceanica* strain.^29^ Yao et al.,^30^ using a rat model, detected 1003 lactylation sites on 469 proteins. Lactylation modification is also prevalent in plants. Zhu et al. identified 927 lactylation sites among 394 proteins in wheat.^31^ Wu et al. detected 215 Kla sites across 138 distinct proteins present in sugarcane.^32^ Meng et al. disclosed the first comprehensive lactylation genetic map for rice, pinpointing 638 lysine‐lactylated locations across 342 proteins found within rice kernels.^33^ According to Shi et al.,^34^ 307 lactylation sites (Kla) were identified on 16 histones throughout the maize genome (Table 1). Collectively, this accumulating evidence establishes lactylation as a phylogenetically conserved PTM.

**TABLE 1:** Research on lactylation modification sites across different species.

Species	Protein quantity	Number of lactylation sites	References
*Trypanosoma brucei*	257	387	^25^
*Toxoplasma gondii*	955	1964	^26^
*Frankliniella occidentalis*	469	1458	^27^
*Carcinogenic streptococcus* mutans	469	1869	^28^
*N. oceanica* strain	379	868	^29^
Rat	469	1003	^30^
Wheat	394	927	^31^
Sugarcane	138	215	^32^
Rice	342	638	^33^
Maize	16	37	^34^

### 2.3 Dynamic regulatory mechanism of lactylation modification

Lactic acid usually exists in three different forms, d‐lactic acid, l‐lactic acid and a mixture of racemic DL‐isomers.^35^ l‐lactic acid, as the main end product of glycolysis, serves as an important metabolite in the glycolysis process and the Cori cycle. Monitoring cell viability and metabolic equilibrium is crucial. l‐lactic acid is known for its important functions in energy production and immune response. Recent study has shown that it can impact the immune system, cardiovascular health and cognitive function. Lactic acid bacteria are the primary producers of d‐lactic acid, which is crucial for addressing issues arising from imbalances in gut flora. The build‐up of d‐lactic acid may represent a potential therapeutic target because of its significant association with the negative interactions between the gastrointestinal system and liver. Recent studies demonstrate that d‐lactic acid influences immunity by altering M2 macrophage activity and the hepatic TME. d‐lactic acid links to oxidative stress, faulty mitochondria and metabolic disturbances, encompassing conditions like diabetes and neurological complications.^36^ DL‐lactic acid contains l‐ and d‐forms, sharing the same characteristics and roles.^37^ DL‐lactic acid and its polymer, poly DL‐lactic acid, have garnered considerable interest in the biomedical field owing to their exceptional biocompatibility.^38^ The initial confirmation concerns to lysine l‐lactylation, a unique protein PTM triggered by l‐lactic acid. l‐type lactate is an energy molecule generated from pyruvate during glycolysis, serves as the precursor. l‐lactate binds with CoA to produce lactyl‐CoA, a high‐energy intermediate whose synthesis is dynamically regulated by the Warburg effect.^39^

Professor Zhao's team^4^ further revealed the transformation of l‐lactic acid into l‐lactyl‐CoA, followed by its acetylation on lysine sites by enzymes like p300. This discovery marked a new phase in understanding lactylation modification mechanisms. Recent studies indicate the presence of techniques for modulating both l‐type and d‐type lactylation. Notably, prevalent deacetylases HDAC1‐3 and SIRT1‐3 can remove both forms, with HDAC3 acting as the most efficient “Eraser” for these modifications.^40^

Expanding research on lactate‐driven epigenetic regulation has led to the identification of enzymatic tools that modulate histone lactylation. These enzymes actively govern the reversible activities of Kla, including writers (which catalyse Kla), erasers (which eliminate Kla) and readers (which identify Kla). Specifically: (1) writers are enzymes that attach lactyl groups to specific protein sites, including P300/CBP, GCN5, and HBO1^41^; (2) erasers are enzymes that remove lactyl groups, including HDAC1‐3, HDAC8 and SIRT1‐3; (3) readers are various proteins with specialized domains that bind and recognize site‐specific epigenetic markers, including the Brg1 protein.^42^, ^43^, ^44^

**FIGURE 1:**
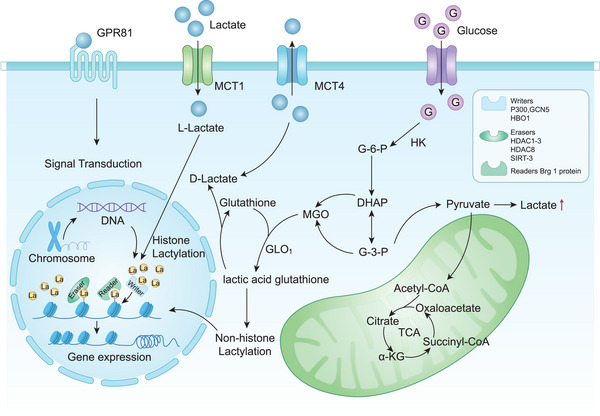
Regulation of lactylation process. L‐lactic acid undergoes lactylation through a complex enzymatic mechanism involving a team effort: writer enzymes (lactylation catalysts), reader enzymes (recognition proteins) and eraser enzymes (DE‐lactylation agents). Lactoylglutathione (LGSH) facilitates the spontaneous transfer of a lactoyl group to lysine residues on proteins without requiring enzyme involvement. This non‐enzymatic lactylation reaction generates d‐lactic acid as a by‐product.

Beyond enzymatic pathways, non‐enzymatic lactylation mechanisms also have play significant roles. Wang et al. conducted pioneering work showing that lysine residues regulated by the deacetylase SIRT5 are susceptible to substantial non‐enzymatic lactylation.^45^ In 2020, Dominique et al. identified a novel non‐enzymatic lactylation mechanism: Methylglyoxal (MGO), a glycolysis by‐product, reacts with glutathione via glyoxalase 1 (GLO1) to form lactoylglutathione (LGSH). The glyoxalase 2 (GLO2) enzyme subsequently breaks down LGSH, freeing glutathione and d‐lactate in the process. A key aspect is the ability of LGSH to autonomously transfer its acyl group to the lysine amino acid in the protein, allowing lactylation modifications to occur independently of the enzyme(Figure 1).^46^

### 2.4 Macrophage polarization process

Currently, two dominant ideas about the origin of macrophages are acknowledged. It is believed that haematopoietic stem cells derived from the bone marrow produce monocytes that then enter the circulation system and spread into different tissues. When monocytes are subjected to certain things, like tissue damage, localized growth factors or pro‐inflammatory cytokines, they turn into tissue‐specific macrophages.^47^ It has been proposed that during embryonic development, embryonic progenitor cells derived from the yolk sac transform into tissue‐resident macrophages, a process driven by liver monocytes. These embryonic cells go through the body and settle in different organs. As the organs grow, they turn into a stable group of macrophages.^48^ These cells engage with mature macrophages derived from bone marrow.

Macrophages are very important for controlling the immune system, and their heightened sensitivity to stimuli has a big effect on how adaptive immune responses are modulated. Plasticity describes macrophages' ability to adapt their phenotype in response to environmental cues. M1 and M2 represent the two primary macrophage subtypes.^49^ Macrophages exhibit two primary polarization states: classically activated M1 and alternatively activated M2 phenotypes.^50^ The M1 subtype emerges in response to Th1‐associated cytokines such as interferon‐gamma (IFN‐γ), tumour necrosis factor‐alpha (TNF‐α) and granulocyte‐macrophage colony‐stimulating factor (GM‐CSF). These immune cells secrete an array of pro‐inflammatory signalling molecules, such as interleukin‐1 beta (IL‐1β), TNF‐α, IL‐1α, IL‐6, IL‐12, IL‐23 and cyclooxygenase‐2 (COX‐2) along with small amounts of IL‐10.^51^ Inducible nitric oxide synthase (iNOS), predominantly in M1 macrophages, is essential for pathogen clearance via nitric oxide (NO) generation. Macrophages, specifically the M1 variety, predominantly tap into glycolysis as their primary energy source, but they also get a boost from the pentose phosphate pathway (PPP), a chopped‐up TCA cycle, and less efficient mitochondrial oxidative phosphorylation (OXPHOS). While glycolysis is the headliner, these supporting metabolic routes kick in to keep cellular operations going strong.^52^ These metabolic pathways support their role in initiating Th1 responses, inducing microbial cell death, promoting inflammation, causing tissue damage and suppressing tumour suppression.^6^ Conversely, cytokines associated with Th2 immunity such as IL‐4, IL‐10, IL‐13 and IL‐33 induce macrophages to enter an M2‐activated state, which in turn produce various anti‐inflammatory mediators such as TGF‐β, IL‐1β, TNF‐α, IL‐6, IL‐10 and IL‐12.^53^ Contrastingly, M2 macrophages refrain from producing nitric oxide. Rather, they up their game by amping up arginase‐1 activity, a molecule that breaks down l‐arginine to combat the cytotoxic effects of NO. This mechanism ultimately supports cell growth by spurring on polyamine production and collagen synthesis.^54^ M2 macrophages exhibit TCA cycle and oxidative phosphorylation‐dependent metabolism, which are essential for their functions in tissue remodelling, immune regulation, Th2 responses, inflammation resolution, parasite clearance and tumour promotion.^8^ The traditional M1/M2 classification system, while providing a basic framework for understanding macrophage function, is overly simplistic and insufficiently represents the dynamic nature and extensive diversity of macrophages within the body.^55^, ^56^ Recent investigations have revealed that macrophages undertake increasingly complex physiological functions within microenvironments. Atherosclerosis exemplifies how stimuli like a high‐fat diet can induce local macrophage proliferation, alongside a phenotypic transformation. In the TME, macrophages that have infiltrated into the tumour mainly show an immunosuppressive state with M2‐like phenotypic characteristics; however, they also encompass M1‐like subpopulations that possess immune‐activating functions. Additionally, macrophages perform distinct physiological functions across various tissues, including involvement in iron cycling in the spleen, synaptic pruning in the brain and the regulation of heat production in brown adipose tissue.^57^

Macrophage polarization represents a dynamic, reversible process modulated by diverse environmental signals, facilitating the transition from M1 to M2 phenotypes. This phenotypic plasticity is orchestrated through complex crosstalk among key signalling pathways, primarily JAK/STAT, PI3K/AKT, JNK and Notch. The polarization of M1 macrophages is primarily triggered by signals such as IFN‐γ and LPS, which activate the PI3K‐AKT‐mTOR‐HIF‐1α signalling cascade. Alongside this, key pathways like JAK‐STAT1, Notch and NF‐κB also play crucial roles in steering macrophages towards the M1 phenotype. The JNK‐STAT pathway regulates IL‐4/IL‐13‐induced M2 macrophage polarization.^58^ The polarization of macrophages depends heavily on hypoxia‐inducible factors (HIFs), with HIF‐1α playing a key role in Th1 cytokine signalling and M1 macrophage activation, while HIF‐2α is more closely tied to M2 macrophage function. When HIF‐2α triggers the expression of Arginase 1 (Arg‐1), it leads to a build‐up of ornithine and suppresses the synthesis of nitric oxide.^59^ Moreover, HIF‐2α enhances mitochondrial oxidative phosphorylation, thereby promoting the M1‐to‐M2 transition, which ultimately attenuates inflammation‐associated pathologies.^60^ This transition ultimately results in a decrease in inflammation‐related illnesses. In the TME, TAMs display both M1 (tumour‐suppressive) and M2 (tumour‐promotive) phenotypes. TAMs in the TME largely operate similarly to M2, contributing to tumour development, angiogenesis, immunosuppression and metastasis.^61^ A well‐established correlation exists between lactate and macrophage polarization. Lactate modulates macrophage metabolism under inflammatory conditions and promotes TAM polarization in cancer. Acting as a ligand for GPR81, lactate activates this receptor's signalling pathway, subsequently influencing metabolism‐related gene expression and facilitating tumour progression.^17^, ^62^ Moreover, lactate plays a key role in shaping macrophage behaviour by fostering the growth of helper T cells and boosting IFNγ production—a process primarily controlled by HIF‐1α activation. Beyond this, it also triggers the release of vascular endothelial growth factor (VEGF) and encourages macrophages to adopt an M2‐like phenotype.^63^

**FIGURE 2:**
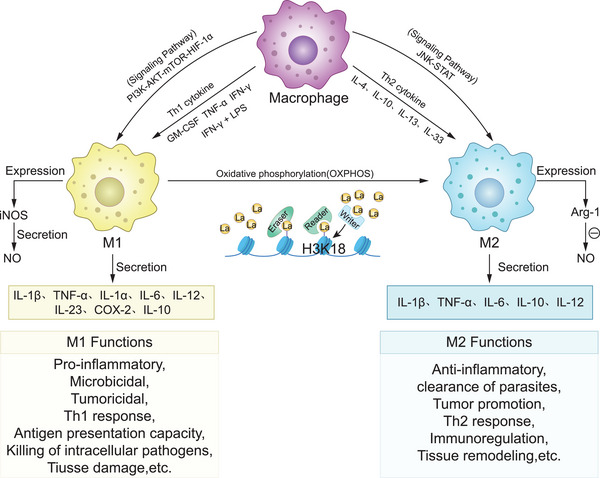
The macrophage activation process. Macrophages come in two distinct types, M1 and M2, which are distinguished by their roles within the immune response. They can switch between these states along various pathways. Furthermore, M1 cells have the ability to transform into M2 cells through a process called lactylation.

Macrophages possess various PTMs that alter their functions and influence immune and inflammatory regulation across different contexts.^64^ Specific modifications involved in macrophage control encompass acetylation, deacetylation, succinylation, malonylation and the recently discovered lactylation. Recent study reveals that lactic acid is essential for the immunological control of macrophages through many pathways. It serves as a metabolic substrate, entering the TCA cycle to form acetyl‐CoA. Acetyl‐CoA is crucial for promoting acetylation of histone H3K27, which leads to increased transcription of Nr4a1 and several other genes involved in downregulating immune responses. As a result, this process dampens the inflammatory response typically seen in macrophages.^65^ Furthermore, lactic acid has led to the emergence of a novel PTM known as lactylation. At its core, this process hinges on triggering an innate “lactate timer” within M1 macrophages through aerobic glycolysis. As M1 macrophages reach full polarization, this biological clock prompts lysine lactylation at histone site H3K18, which in turn activates genes associated with M2‐like macrophage characteristics (Figure 2).^4^ While investigations have established a connection between Arg1 production and histone lactylation,^66^ other findings suggest that histone lactylation operates independently from the gene expression patterns seen in fully activated M2 macrophages. Further research is recommended in this domain as the specific procedures remain not fully understood.

## 3 MODULATION OF MACROPHAGE ACTIVITY IN TUMOURS BY LACTYLATION MODIFICATION

Histone lactylation profoundly impacts tumour biology, affecting carcinogenesis and progression through multiple mechanisms. Elevated lactate levels within the TME, often termed the glycolytic switch or Warburg effect,^67^ serves as an indicator of reprogrammed cellular metabolism. Critically, this modification is highly sensitive to both endogenous and external lactate levels.^4^ During glycolysis, lactate and acetyl‐CoA concentrations collectively dictate the cell's outcome, driving cells towards cancer and promoting carcinogenesis.^18^ Lactylation influences gene expression in cancer cells and neighbouring TME components.^68^ For example, it may enhance TAMs infiltration into the TME^69^ or disrupt intercellular interactions between TAMs and neutrophils, thereby impairing antigen‐specific immunity against invasive cancers.^70^ Moreover, lactylation critically alters non‐cancerous cell behaviour in the TME, fostering tumour growth and facilitating immune escape.^71^ It also plays a pivotal role in controlling DNA repair processes within cancer cells while governing dysfunctional glucose metabolism. Moreover, changes in the lactylation patterns of non‐histone proteins can directly impact both chemotherapy resistance and malignant proliferation.^72^

### 3.1 Increased tumour cell proliferation

Complex interactions exist between the TME and tumour growth.^73^ Histone lactylation influences tumour biology by modifying gene expression inside the TME through its involvement in the metabolic‐epigenetic‐transcriptional axis, with particular focus on the lactylation at the H3K18 site.^74^

In lung cancer, infiltrating macrophages undergo M2‐like polarization (TAM phenotype), facilitating tumour cell proliferation, invasion and metastasis.^75^ Lactate is a central driver of TAM polarization within the TME, reflecting the heightened lactate and glucose consumption characteristic of lung malignancies.^76^ Crucially, lactate stabilizes HIF‐1α and induces H3K18la, thereby promoting M2‐like gene expression and driving lung cancer progression.^77^ Furthermore, elevated lactylation correlates with differential expression of Solute Carrier Family 2, Member 1 (SLC2A1), also known as GLUT1, predominantly in SPP1+ macrophages. When HIF‐1α and glucose transporter‐1 (GLUT1) co‐localize in hypoxic conditions, lung cancer becomes more advanced, with HIF‐1α driving macrophages to polarize towards the M2 phenotype.^78^

Gastric cancer, the most prevalent digestive system malignancy, exemplifies how lactate promotes H3K18 lactylation (H3K18la), subsequently boosting the transcription of vascular cell adhesion molecule‐1 (VCAM1). This process then triggers the activation of the AKT‐mTOR signalling cascade, leading to an increased production of CXCL1.^79^ Lactylation promotes M2 macrophage recruitment and activation, driving tumour growth, spread and epithelial‐mesenchymal transition (EMT).^80^ GLUT3 overexpression in gastric cancer tissues and immune cells (including regulatory T cells [Tregs] and macrophages) promotes invasiveness. Gastric cancer's advancement can be modulated by GLUT3 reduction, inhibiting LDHA, l‐lactate and the lactylation of histone marks H3K9, H3K18 and H3K56.^81^

In bladder cancer, H3K18 lactylation is equally significant. CircXRN2, identified via RIP and circRNA sequencing, interacts with SPOP to inhibit LATS1 ubiquitination and degradation. This triggers the Hippo pathway, suppressing H3K18 lactylation and consequently downregulating glycolysis and lactate production. H3K18 lactylation acts oncogenically by enhancing lipocalin 2 (LCN2) oncogene expression; inhibiting this mechanism can impede cancer metastasis.^82^

The colorectal cancer (CRC) TME displays elevated lactate levels, leading to H3K18 lactylation, thereby repressing RARγ gene expression in macrophages.^83^ Activating the STAT3 signalling pathway in CRC cells enables macrophages to drive carcinogenesis. What is more, the presence of H3K18la in tumour‐infiltrating myeloid cells ramps up METTL3 production. This triggers the m6A‐YTHDF1 signalling cascade, which in turn supercharges Jak1 protein synthesis. The resulting STAT3 activation could fuel unchecked tumour growth.^84^ H3K18la plays a multifaceted role in driving tumour progression by upregulating RUBCNL/Pacer expression. It facilitates autophagosome maturation through its interaction with BECN1 and orchestrates the assembly and activity of the class III phosphatidylinositol 3‐kinase complex. These mechanisms collectively contribute to enhanced neoplastic proliferation. This is crucial for hypoxic cancer cells' survival and proliferation.^85^ Furthermore, GPR37, upon activation, triggers the Hippo pathway, thereby boosting glycolysis and amplifying LDHA levels. This enhances H3K18la lactylation and upregulates CXCL1 and CXCL5, influencing tumour metabolism and patient outcomes.^86^ The recently discovered lactylation modification drives M1 macrophage polarization and accelerates colon cancer development. This process is functionally tied to PCSK9, which influences tumour cell EMT and the PI3K/AKT signalling cascade by suppressing lactate levels, histone lactylation and macrophage migration inhibitory factor (MIF) activity.^87^

Recent studies reveal that heightened H3K18la concentrations in ocular melanoma tumours correlate with unfavourable patient outcomes. This epigenetic marker exerts its effects by modulating YTHDF2, a key reader protein that targets m6A‐marked PER1 and TP53 transcripts for destruction. By facilitating the breakdown of these tumour‐suppressing mRNAs, the H3K18la‐YTHDF2 axis drives the aggressive advancement of ocular melanoma.^88^ Studies have shown that eliminating m1A methylation from SP100A enhances H3K18 lactylation, increasing ALKBH3 expression. ALKBH3 disrupts tumour‐suppressive PML body formation, facilitating ocular melanoma malignant transformation.^89^ In ovarian cancer, high H3K18la levels predict poor prognosis and represent a potential therapeutic target.^90^ Tumour‐derived lactate activates macrophage Gpr132, acidifying the TME and polarizing TAMs toward M2 phenotype. Lactate upregulates M2‐like genes via H3K18 lactylation to promote tumourigenesis and metastasis.^91^ Similarly, in cervical cancer, MCT1‐mediated lactate transfer to macrophages enhances GPD2 transcription via H3K18 lactylation, inducing M2 polarization and malignant progression.^92^

Moreover, changes in lactate levels can activate the body's inherent positive feedback mechanisms, promote carcinogenesis and enhance tumour cell proliferation and spread. Pancreatic carcinoma (PC) ranks among the most aggressive and fatal malignancies affecting the gastrointestinal tract.^93^ The increased glycolysis in pancreatic ductal adenocarcinoma leads to heightened H3K18 lactylation and lactate production, which promotes the transcription of BUB1B and TTK. TTK and BUB1B increase lactate levels, modulated by P300, forming a positive feedback loop that drives oncogenesis.^94^ Moreover, the CCTC‐binding factor (CTCF) engages with HNRNPU via an FLG‐AS1‐mediated pathway, facilitating the recruitment of EP300 to upregulate the m6A reader IGF2BP2. Beyond boosting histone lactylation and fuelling pancreatic cancer cell growth, IGF2BP2 also promotes the M2 polarization of TAMs, playing a key role in the progression of pancreatic cancer.^95^ Meanwhile, renal cell carcinoma—the predominant form of urothelial cancer—proves lethal in roughly 30%–40% of cases.^96^ Clear cell renal cell carcinoma pathogenesis involves VHL inactivation, causing H3K18 histone lactylation, which activates PDGFRB transcription. This establishes a lactylation‐PDGFRB positive feedback loop that accelerates tumour growth.^97^ In breast cancer cells, KCNK1 interacts with LDHA activation, enhancing glycolysis and lactate synthesis. LDHA initiates a positive feedback loop, reducing tumour cell stiffness and adhesion through H3K18 histone lactylation. This activation increases the expression of numerous genes in the downstream and enhances LDHA, fostering breast cancer's growth, penetration and spread.^98^ Endometrial cancer exhibits a significant rise in histone lactylation, with H3K18 lactylation being especially prominent. This modification drives tumour progression by upregulating USP39 expression, which in turn stimulates the PI3K/AKT/HIF‐1α signalling cascade and boosts glycolytic activity. Essentially, H3K18la fuels cancer cell proliferation through these interconnected metabolic and molecular mechanisms.^99^

### 3.2 Facilitation of immune evasion

Lactate‐dependent immune evasion critically involves macrophages. In neuroblastoma (NB), M2‐like TAMs enable progression and immune escape within the TME.^100^ Hexokinase‐3 (HK3), a crucial glycolytic enzyme in neuroblastoma, regulates the PI3K/AKT‐CXCL14 axis, which attracts and polarizes M2‐like macrophages, influencing directly the malignant characteristics of tumour cells. Histone lactylation and lactate synthesis are likewise affected. Furthermore, the modification of HK3 can alter tumour cell behaviour in M2 tumour‐associated macrophages.^101^ In non–small cell lung cancer, histone H3K18 lactylation activates the POM121/MYC/PD‐L1 signalling axis, giving tumour cells a powerful edge in dodging immune detection. Mouse model research demonstrated that boosting CD8+ T cell‐mediated anti‐tumour immunity while suppressing lactate‐induced H3K18 lactylation effectively blocked non–small cell lung cancer's immune escape mechanisms.^102^

Liver cancer cells and macrophage precursors share a common ancestral origin, influencing macrophages phenotypic switching and exacerbating disease progression.^103^ Lactate levels shoot up inside and outside the cell because of a dynamic dance between the SRSF10, a key member of the SRSF family known for its serine and arginine residues, and the 3′‐untranslated region of MYB. This lactate triggers a biochemical coup, leading to lactylation of histone H3K18, which in turn boosts SRSF10 production and gets the transcription gears rolling. This subsequently enhances the activity of tumour‐associated precursor macrophages. Additionally, alterations in lactate concentrations trigger the polarization of M2 macrophages, impacting tumour immune suppression.^104^ The liver is the primary metastatic site in CRC patients. Analysing macrophages and their spatial distribution within the TME provides crucial therapeutic insights.^105^ Intestinal microbiota dysbiosis activates immune cells, particularly macrophages, triggering increased endotoxin release and cytokine production. This compromises the self‐healing capacity of colonic epithelial cells and facilitates tumour cell invasion, metastasis and proliferation.^106^ New research reveals that lactate produced by *Escherichia coli* disrupts mitochondrial antiviral signalling and NF‐κB pathways through RIG‐I lactylation at the K852 site. This mechanism promotes the polarization of M2 macrophages and the differentiation of Tregs, ultimately accelerating the spread of CRC to the liver. Essentially, bacterial lactate hijacks immune regulation, creating a tumour‐friendly environment that fuels metastasis.^107^ Additionally, histone lactylation on the LINC00152 promoter suppresses *Salmonella* invasion and inflammatory responses induced by gut bacterial LPS. The increase in histone H4K8la within macrophages weakens YY1‐mediated repression of LINC00152, leading to its upregulation. As a consequence, elevated LINC00152 expression fuels the spread of CRC to the liver by amplifying tumour cell motility and aggressiveness.^108^

The establishment of lactylation‐related models in gastric cancer has shown significant promise in enhancing patient responses to immunotherapy and targeted therapies, as well as in predicting disease progression. Zhang et al. crafted a framework centred on genes linked to hypoxia‐glycolysis‐lactylation pathways, proven to accurately predict GC patients’ survival outcomes and drug responsiveness. This model accounts for substantial differences in immune infiltration, especially between M1 macrophages and CD8+T cells.^109^ Yang et al. achieved analogous results by employing a lactylation model to forecast immune evasion and malignant proliferation in GC. High lactylation scores are associated with increased genetic instability and significant immune cell infiltration, especially macrophages.^110^

Since its inception, researchers’ interest in utilizing lactylation‐related models to predict tumour growth in gastric cancer has been growing, generating much enthusiasm among scientists to apply these models to various types of cancer. Lactylation influences tumour immune function, macrophage infiltration and the progression of diffuse large B‐cell lymphoma. It also diminishes sensitivity to standard therapeutic agents and enhances treatment resistance. Consequently, genes exhibiting diverse levels of lactylation are crucial for treatment efficacy. Zhang et al. devised a lactylation‐based risk score model to evaluate the risk associated with these genes.^111^ To predict the prognosis of patients with prostate cancer (PCa), Pan et al. created a unique lactylation‐related predictive algorithm. This approach offers novel insights into personalized therapeutics and demonstrates robust efficacy in predicting disease‐free survival.^112^ Lactylation models also exist in hepatocellular carcinoma and nasopharyngeal carcinoma, and can be used to predict tumour occurrence, development and prognosis.^113^, ^114^ As research advances, lactylation predictive models are continuously being developed. Yang et al. constructed two distinct neural network frameworks using natural language processing to improve Kla prediction efficacy: the embedding‐based feature fusion model EBFF‐Kla and the attention‐based feature fusion model ABFF‐Kla. These models consider the impact of 3D structures and protein sequences on Kla sites.^115^ Further research is required to identify specific lactylation‐involved genes and clarify the precise role of these models in disease treatment.

Macrophages significantly contribute to the progression of prostate cancer, a disease experiencing an increasing prevalence.^116^ In PTEN/p53‐deficient prostate cancer, the PI3K, MEK and Wnt/β‐catenin signalling pathways work together to facilitate tumour immune evasion. They do this via lactate production and histone lactylation (H3K18lac), respectively. Research indicates that blocking these pathways may reduce lactate productions, thereby decreasing H3K18lac levels and activating TAMs to curb tumour growth.^117^ Evodiamine, a naturally occurring alkaloid from Evodia rutaecarpa fruit, specifically targets the histone H3K18 lactylation site. In PCa, H3K18lac suppresses lactate‐induced lactylation of HIF‐1α, which in turn prevents Sema3A from reducing PD‐L1 expression, immune cell infiltration, cellular adhesion and angiogenesis. Moreover, evodiamine significantly reduces the survival of PCa cells, potentially enhancing the effectiveness of immunotherapy or anti‐angiogenic therapies.^118^

In glioblastoma, glioma stem cells co‐cultured with CD4^+^ T cells and macrophages, as well as cells exposed to lactate, exhibit elevated levels of uCD39, CD73 and CCR8. Intracellular lactate directly activates these gene promoters through histone H3K18 lactylation. Altering the glycolytic characteristics of tumour stem cells and limiting lactate production reduces tumour‐infiltrating CAR‐Treg cells and alleviates the immunosuppressive TME.^119^ Glioblastoma exhibits profound oxygen deficiency, resulting in macrophage build‐up within oxygen‐starved areas.^120^ Furthermore, lactate accumulating in glial cells due to hypoxia is taken up by macrophages. This process initiates M2 polarization via the MCT1/H3K18La/TNFSF9 pathway, accelerating glioma malignant progression.^121^ Histone lactylation driven by intracellular lactate plays a key role in amplifying the immunosuppressive properties of monocyte‐derived macrophages (MDMs) within glioblastoma microenvironments. This PTM triggers elevated IL‐10 secretion from MDMs, effectively dampening T cell function and creating an immune‐evasive niche. Furthermore, the metabolic reprogramming induced by lactylation promotes glucose metabolism, steering macrophage polarization toward the pro‐tumoural M2 phenotype while simultaneously activating the PERK‐ATF4 signalling axis. These interconnected mechanisms collectively supercharge MDMs' capacity to undermine anti‐tumour immunity.^122^

### 3.3 Promoting tumour drug resistance

Extensive recent research links lactylation alterations to tumour therapy resistance, particularly concerning DNA repair and chemoresistance. As per Li et al., NBS1 lactylation propelled by lactate boosts homologous recombination‐mediated DNA damage restoration, boosting tumour cells’ DNA damage repair capacity. This accelerated repair post‐radio chemotherapy reduces treatment efficacy and causes resistance,^123^ thereby identifying lactylation as an innovative focus for overcoming cancer medication resistance. Current research shows the CBP acetyltransferase lactylates the essential HR protein MRE11 at position K673, increasing its DNA affinity and repair ability. This enhancement confers tumour resistance to PARP inhibitors and platinum‐based treatments.^124^

**FIGURE 3:**
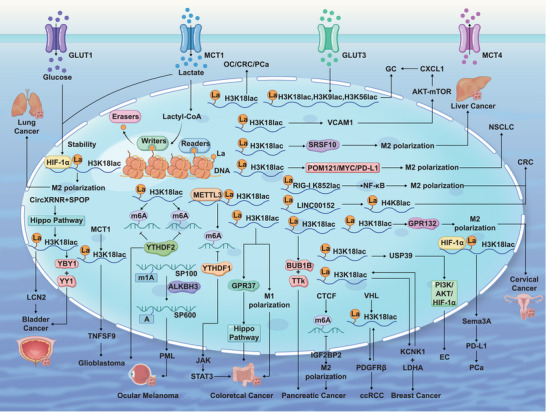
The role of lactylation in cancer development. The advancement of tumours is affected by variations in lactate and lactylation, which modulate abnormalities in macrophage phenotype. These alterations transpire during tumourigenesis. ccRCC, clear cell renal cell carcinoma; CRC, colorectal cancer; EC, endometrial carcinoma; GC, gastric cancer; NSCLC, non–small cell lung cancer; OC, ovarian cancer; PCa, prostate cancer.

In CRC, bevacizumab resistance is significantly attributed to histone lactylation. Elevated H3K18 lactylation levels may influence autophagy and metabolic reprogramming, potentially contributing to treatment resistance and a poor prognosis in CRC patients. Inhibiting H3K18 lactylation could enhance bevacizumab's therapeutic efficacy in CRC treatment.^85^ In bladder cancer, histone lactylation (H3K18la) strengthens promoter regions and promotes target gene transcription. H3K18la drives key transcription factors YBX1 and YY1, which are crucial for cisplatin resistance. Thus, targeting H3K18la can restore cisplatin sensitivity in resistant epithelial cells (Table 2).^125^ Given the limited research on this topic, scholars may explore and devise novel strategies to counteract tumour treatment resistance. Researchers should use these insights to explore lactylation's impact on treatment resistance, aiding in the discovery of novel drug targets and strategies (Figure 3).

**TABLE 2:** Relationships between histone lactylation and the study of different types of tumours.

Cancer type	Cell type	Lactylation site	Affiliated molecules or pathways	Effect	References
Lung cancer	TAMs	H3K18	HIF‐1α	Promotion of tumourigenesis and polarization to M2 type	^77^
	Macrophage		POM121/MYC/PD‐L1 pathway	Promotion of tumour cells immune escape	^102^
Liver cancer	M2 Macrophage	H3K18	SRSF10, MYB	Promotion of tumourigenesis and polarization to M2 type	^104^
Gastric cancer	M2 macrophage	H3K18	VCAM1 AKT‐mTOR pathway	Promotion of tumour cell proliferation, metastasis and transformation of EMT	^80^
	M1 macrophage CD8^+^ T		HGLRGs	Enhancement of the efficacy of immunotherapy	^109^
	Immune cell			Predicting tumour progression	^110^
	Macrophage, Tregs	H3K18, H3K9, H3K56	GLUT3, LDHA	Predicting tumour progression	^81^
Colorectal cancer	Macrophage	H3K18	PI3K/AKT pathway	Promotion of tumourigenesis	^87^
			RARγ, IL‐6 STAT3 pathway	Promotion of tumourigenesis	^83^
	TIMs		METTL3 m6A‐YTHDF1pathway	Promotion of tumourigenesis	^84^
	Tumour cell		RUBCNL/Pacer	Promotion of autophagosome maturation	^85^
	Macrophage	H4K8	LINC00152	Promotion of tumourigenesis	^63^
	Macrophage	RIG‐IK852	MAVS, NF‐KB	Promotion of colorectal liver metastasis	^107^
	Macrophage	H3K18	CXCL1, CXCL5 Hippo pathway	Promotion of colorectal liver metastasis	^86^
Pancreatic cancer	Macrophage TAMs	H3K18	TTK, BUB1B CTCF, IGF2BP2	Promotion of tumourigenesis Promotion of tumourigenesis and polarization to M2 type	^94^ ^95^
Renal cell carcinoma	Tumour cell	H3K18	VHL, PDGFRβ	Promotion of tumour proliferation	^97^
Bladder cancer	Tumour cell	H3K18	CircXRN2, LATS1, LCN2 Hippo pathway	Promotion of glycolysis	^82^
	Anti‐cisplatin epithelial cells		YBX1 and YY1	Promotion of cisplatin resistance	^125^
Prostatic cancer	TAMs	H3K18	Wnt/β‐catenin pathway	Promotion of tumour growth	^117^
	Macrophage	H3K18 HIF‐1α	Sema3A, PD‐L1	Promotion of tumour angiogenesis	^118^
Breast cancer	Macrophage	H3K18	LDHA, KCNK1	Promotion of tumour proliferation, metastasis and invasion	^98^
Endometrial cancer	Macrophage	H3K18	USP39 PI3K/AKT/HIF‐1α pathway	Promotion of tumourigenesis	^99^
Ovarian cancer	TAMs	H3K18	Gpr132, CCL18	Promotion of tumourigenesis and polarization to M2 type	^91^ ^91^
Cervical cancer	Macrophage	H3K18	GPD2	Promotion of tumourigenesis and polarization to M2 type	^92^
Melanoma	Macrophage	H3K18	YTHDF2, PER1 and TP53	Promotion of tumourigenesis	^88^
			ALKBH3, SP100A		^77^
Glioblastoma	CD4^+^ T, CRT macrophage	H3K18	uCD39, CD73 and CCR8	Reduction of CAR‐T therapy	^119^
	MDMs		IL‐10 PERK‐ATF4 pathway	Promotion of the enhancement of immune suppressive activity of MDMs	^122^
	Macrophage		MCT1/H3K18La/TNFSF9 pathway	Promotion of tumourigenesis and polarization to M2 type	^121^
Neuroblastoma	TAMs		PI3K/AKT‐CXCL14	Promotion of tumourigenesis and polarization to M2 type	^101^

## 4 MODULATION OF MACROPHAGE ACTIVITY IN INFLAMMATORY DISEASES BY LACTYLATION MODIFICATION

Lactylation is essential in controlling inflammation. Its mechanisms of action are not limited to enzyme‐catalysed pathways, as non‐enzymatic modifications also hold significant biological importance. Relevant studies indicate that S‐D‐lactoylglutathione (SLG)‐mediated non‐enzymatic d‐lactylation can regulate inflammatory processes by constructing an immunometabolic feedback loop. Significantly, GLO2, acting as a pivotal regulatory hub in this metabolic cascade, could emerge as an innovative therapeutic focus for treating inflammatory disorders via targeted modulation.^126^ Given the pivotal role demonstrated by lactylation modification in modulating inflammatory responses, it has attracted increasing attention in the academic community, particularly regarding its profound effects on macrophages—a critical type of immune cell.^127^

KLA plays a pivotal role in regulating the inflammatory cascade. It fine‐tunes the activity of inflammatory genes within activated macrophages, driving their transition from the aggressive M1 state to the reparative M2 phenotype. This molecular switch is crucial for resolving inflammation and restoring tissue homeostasis. TRIM33, a bromodomain‐containing a protein has been identified as a new reader of histone lactylation and is thought to link histone lactylation to macrophage polarization mechanisms. Macrophage phenotype, influenced by various conditions, mainly determines their functional impact. Precisely controlling macrophage activation states to reduce inflammation is an effective therapeutic strategy for inflammatory diseases.^128^

Macrophages become activated during an inflammatory reaction upon exposure to LPS, resulting in heightened lactate generation and enhanced glycolytic activity.^129^ Lactate stimulates Kla, increasing tissue repair‐related gene expression. The gene expression profiles associated with active M2 macrophages and Kla are clearly delineated, with Arg1 expression demonstrating a notable association between the two conditions. The time‐dependent stimulation of Arg1 expression by LPS requires the autocrine‐paracrine release of IL6, the IL6 receptor and subsequent signalling via the Stat3 pathway, all of which are essential components.^66^ Mitochondrial shape directly affects macrophage phenotype. Macrophages with fragmented mitochondria exhibit an enhanced inflammatory response to LPS, evidenced by increased expression of the pro‐catabolic mediator Arg1 and improved phagocytic capabilities. Increased intracellular lactate, histone lactylation and the emergence of an M2 profile are linked to mitochondrial fragmentation.^124^ Each component critically influences inflammation resolution.

### 4.1 The role in autoimmune inflammation

#### 4.1.1 Role in atherosclerosis

Atherosclerosis, or AS, is a condition linked to inflammation and autoimmune responses. It is marked by an imbalance in the way macrophages, a type of white blood cell, are polarized, which hampers the body's ability to resolve inflammation and keeps chronic inflammation going strong.^130^ Emerging evidence demonstrates that macrophage reprogramming is modulated by diverse epigenetic modifications and their regulatory mechanisms, including DNA methylation, histone acetylation and the recently identified lactylation.^131^

Crucially, histone lactylation in macrophages—a process mechanistically linked to atherosclerosis pathogenesis—requires the lactate efflux transporter MCT4. Notably, the absence of MCT4 triggers histone H3K18 lactylation, which amplifies the expression of genes linked to anti‐inflammatory responses and the TCA cycle. This molecular mechanism plays a pivotal role in driving the shift from M1 to M2 macrophage polarization,^132^ thereby initiating tissue repair mechanisms and restoring homeostatic functions. Notably, the K271 residue emerges as a critical regulatory site in atherosclerosis progression. Methyl‐CpG binding protein 2 (MeCP2), as a key epigenetic regulator of macrophages' response to environmental stimuli, undergoes exercise‐induced lactic acid modification at the K271 locus. The modifications he made enhanced plaque stability, reduced atherosclerosis pathology and prompted macrophages to polarize towards the repair M2 phenotype by inhibiting the expression of RUNX1, a molecular determinant of disease severity.^133^ Alongside atherosclerosis, exercise‐induced lactylation modification may serve as a regulatory mechanism that connects systemic lactate flow with local epigenetic reprogramming. Lactic acid serves as both a terminal product of glycolysis and a metabolic messenger, which cells can actively perceive and convert into epigenetic signals. The study found that the lactylation modification of MeCP2 is involved in the polarization of macrophages in liver fibrosis and neuroinflammation, indicating a potential cross‐organ conservative lactylation modification.^134^ Varying degrees of epigenetic modifications influenced by different exercise intensities suggest the utility of customized training programmes or lactate‐mimicking compounds, such as MCT1 activators and GCN5 selective modulators. These interventions may enhance lactylation modification, potentially broadening the cardiovascular protective benefits of exercise to encompass metabolism, tumours and neurodegenerative diseases.

Moreover, persistent inflammatory damage prompts vascular smooth muscle cells (VSMCs) to switch identities, transforming into macrophage‐like cells—a phenomenon closely linked to the development of atherosclerosis and changes in blood vessel structure. At the molecular level, the activation of the SOX10 gene through a process involving phosphorylation‐driven lactylation directs the reprogramming of gene expression that drives this trans differentiation of VSMCs. This pathway promotes pyroptotic expansion and macrophage‐like VSMC generation while perpetuating vascular inflammation, ultimately driving atherogenesis.^135^

#### 4.1.2 Role in rheumatoid arthritis

Rheumatoid arthritis (RA) is a persistent inflammatory disorder characterized by joint lining inflammation, abnormal tissue growth and pannus development. The onset of RA is characterized by significant macrophage infiltration into the synovium.^136^ Maintaining the appropriate balance between different macrophage polarization states is crucial to eliminating inflammation, but studies have shown that the balance between M1 and M2 macrophage populations is disrupted in patients with RA.^137^

In the final stage of M1 macrophage polarization, increased lactylation level of histone H3K18 leads to changes in the activation of M2‐like gene expression. Recent research has highlighted the important role of lactic acid modifications on non‐histone proteins in inflammatory diseases. When lactylation occurs at the K62 position, it prevents the conversion of pyruvate kinase M2 (PKM2) from its tetrameric form to its dimeric form, limiting its entry into the cell nucleus and reducing glycolytic activity. Together, these effects direct the transition of LPS‐activated macrophages to an M2‐like phenotype.^138^ The results suggest that macrophages release lactic acid through the MCT4 transporter to maintain high levels of glycolysis, while fibroblast‐like synoviocytes adopt a pro‐inflammatory phenotype by assimilating lactate via MCT4.^139^ In the course of inflammation, a heightened presence of M1 macrophages generates significant quantities of pro‐inflammatory factors and chemokines, thereby exacerbating synovial inflammation, facilitating pannus formation and contributing to bone deterioration, which affects the manifestations of RA.

The splicing regulator RBM25 emerges as a critical lactylation‐dependent modulator in RA progression. Clinically, RBM25 suppresses macrophage‐induced inflammatory responses and IL‐1β secretion, with its deficiency associated with autoimmune tissue inflammation and inflammatory senescence. RBM25 modulates the exclusion of exon 14 from ATP citrate lyase (Acly) in a manner reliant on protein lactylation modification (K918/995 sites), altering the glycolytic response and inflammatory gene transcription programme of macrophages, therefore impacting the progression of RA disease.^140^

#### 4.1.3 Role in lupus nephritis

Systemic lupus erythematosus is a persistent autoimmune disease that affects multiple organs throughout the body. Lupus nephritis is a frequent and serious renal complication of systemic lupus erythematosus. Glycolysis, lactate metabolism and protein lactylation are implicated in lupus nephritis pathogenesis.^141^ Sun et al., using two machine learning methods, identified eight key genes and three lactate‐metabolism‐related biomarkers (COQ2, COQ4 and NDUFV1), while assessing lactate indicators in lupus nephritis to explore potential mechanisms. They also found links between macrophage M0, Tregs and these three biomarkers^142^ (Table 3).

**TABLE 3:** Analysis of histone lactylation and autoimmune inflammatory diseases research.

Disease type	Cell type	Lactylation site	Affiliated molecules or pathways	Effect	References
Atherosclerosis	M1 Macrophage	H3K18 K271	MeCP2, RUNX1	Promote the formation of arteriosclerosis and polarization to M2 type	^132^ ^133^ ^135^
Rheumatoid arthritis	Macrophage	H3K18 K62 K918/995	PKM2, FLSs, MCT4 RBM25	Polarization to M2 type	^138^ ^139^ ^140^
Systemic lupus erythematosus	M0 Macrophage Tregs		COQ2, COQ4 and NDUFV1	Promotion of the occurrence of lupus nephritis	^142^

### 4.2 Role in sepsis and infections

#### 4.2.1 Role in sepsis

The pathophysiological mechanisms of sepsis involve stimulation of neutrophils and monocytes/macrophages, as well as the continued secretion of pro‐inflammatory substances and the initiation of the coagulation process.^143^ Emerging evidence indicates that post‐translational lactylation modifications regulate macrophage functions during sepsis progression. High serum lactic acid concentrations are associated with an increased risk of death in patients with sepsis, where lactic acidosis and acute lung injury (ALI) are common complications. In the context of sepsis‐induced ALI, lactic acidosis promotes lactylation and secretion of cold‐induced RNA‐binding protein by macrophages. This process promotes the development of ZBP1‐dependent PANOPSIS in pulmonary vascular endothelial cells, thereby exacerbating the severity of ALI.^144^

Furthermore, histone H3K18 lactylation demonstrates critical regulatory roles in sepsis. H3K18la may serve as a prognostic biomarker for infection status and critical illness severity. This epigenetic modification significantly modulates macrophage anti‐inflammatory responses through H3K18la‐mediated mechanisms, including enhanced IL‐10 production and upregulated Arg1 expression in septic conditions.^145^ Severity and mortality of sepsis are linked to HMGB1 and circulating lactate levels. MCTs allow macrophages to absorb extracellular lactate, and a p300/cbp‐dependent mechanism allows them to increase the lactylation of HMGB1.^21^ Lactylation‐modified genes including S100A11 and CCNA2 have been implicated in sepsis pathogenesis, showing potential as diagnostic and prognostic biomarkers. Single‐cell RNA sequencing analyses reveal significant expression of these critical genes in monocytes/macrophages, T lymphocytes and B lymphocytes.^146^

#### 4.2.2 Role in bacterial infections

During infections with methicillin‐resistant *Staphylococcus aureus* (MRSA), the Type I IFN signalling pathway within macrophages is modulated to limit lactate production, consequently affecting histone H3K18 lactylation. This regulatory mechanism prolongs MRSA‐induced iNOS expression, exacerbates inflammatory responses and promotes bacterial dissemination.^147^ In related investigations, methyl methane sulfonate has been shown to upregulate metabolic pathways, particularly glycolysis, in peritoneal macrophages during MRSA infection's administration enhances the expression of H3K18 lactylation‐specific target genes, including Arg1, which promotes STAT3 phosphorylation and modulates the polarization towards M2 macrophages, while suppressing M1 macrophage‐associated gene expression.^148^

### 4.3 Role in neuroinflammation

Microglia and non‐neuronal macrophages are increasingly recognized as playing pivotal roles in CNS development, homeostasis and disease, with significant implications for neuroinflammation.^149^ Lactylation modification at the p53 site may help reduce neuroinflammatory damage by inhibiting lactate production during LPS‐induced macrophage pro‐inflammatory activation and promoting the upregulation of nuclear p65 protein phosphorylation levels in BV2 cells, an immortalized mouse microglia cell line.^150^ Spinal cord injury (SCI) is a severe central nervous system disorder that triggers complex neuroinflammatory responses. Following SCI, spinal cord lactate levels rise significantly, leading to increased lactylation in microglia and elevated H4K12la levels. This in turn stimulates PD‐1 transcription in microglia.^151^ Furthermore, H4K12la also can promote Spp1 transcription through reprogrammed microglia post‐injury, contributing to SCI recovery.^152^

### 4.4 Role in chronic inflammatory diseases

#### 4.4.1 Role in ulcerative colitis

Engineered *Saccharomyces cerevisiae* capable of producing lactate from glucose has been utilized to reduce the expression of pro‐inflammatory cytokines and modulate macrophage polarization in ulcerative colitis, both in vitro and in vivo. The produced lactate mitigates colitis symptoms by preventing macrophage pyroptosis, altering gut microbiota, promoting histone H3K9 acetylation and H3K18 lactylation.^153^ Furthermore, Gegen Qinlian Decoction, a traditional Chinese medicine, has demonstrated efficacy in altering macrophage polarization and the course of ulcerative colitis. In colitis mouse models, it diminishes tissue damage and colon shortening, lowers serum LDH levels and restrains lactate production, thereby controlling histone lactylation. Its mechanisms involve suppressing pan‐lactylation and specific lactylation sites (H3K18la, H3K23la, H4K8la, H4K12la). Additionally, by modulating macrophage polarization, Gegen Qinlian Decoction decreases M1 macrophages and increases M2 macrophages in colonic tissue, exerting anti‐inflammatory effects.^154^

#### 4.4.2 Role in asthma

Asthma is recognized as a common chronic inflammatory disease, and research suggests that glycolytic reprogramming may help asthma management.^155^ Chen's team found Dexamethasone (DEX) improves asthma management by suppressing the Hif‐1α‐glycolysis‐lactate pathway, affecting protein lactylation. They exposed animals to ovalbumin, an airborne allergen and then stimulated the human macrophage cell line THP‐1 after treatment with DEX. DEX inhibits the ovalbumin‐induced Hif‐1α‐glycolytic pathway, thereby reducing lactate levels. Key glycolytic enzymes were significantly co‐localized with F4/80‐positive macrophages in the lungs of asthmatic mice, indicating a metabolic transition to glycolysis in lung macrophages during asthma.^156^

## 5 MODULATION OF MACROPHAGE ACTIVITY IN ORGAN DAMAGE AND TISSUE REPAIR BY LACTYLATION MODIFICATION

Lactylation may contribute to organ injury healing and the onset and progression of pathological fibrosis through multiple mechanisms. This alteration fosters a conducive microenvironment for tissue regeneration by regulating immune cell activity and modulating the inflammatory response.^157^ Nonetheless, when damage endures, acute and chronic inflammation resulting from cell necrosis, infection or toxic agents may hinder the repair process, culminating in fibrosis marked by excessive extracellular matrix deposition. Fibrosis, a prevalent final pathological process in numerous chronic inflammatory disorders, arises from sustained necrosis of parenchymal cells due to inflammation, and its advancement ultimately results in organ failure and potentially mortality.^158^

### 5.1 Role in acute organ injury

#### 5.1.1 Role in hepatic injury

In the pathophysiology of liver injury and its healing process, macrophages play an indispensable role.^159^ When the liver is damaged, liver cells quickly undergo cell death, triggering iron death and triggering an inflammatory response. Iron death also activates the NF‐κB signalling pathway, which promotes the polarization of M1 macrophages and ultimately leads to an intensification of the inflammatory response.^160^ Lactylation modification may affect liver damage by regulating macrophage polarization. Salviolic acid B (Sal B) is the main water‐soluble bioactive substance extracted from the roots of Salvia Miltiorhiza Bunge and is an important factor in liver damage. In M1 macrophages, Sal B reduces levels of lactylation‐modified histone H3K18la near the promoter regions of the LDHA gene and the inflammation‐related genes NLRP3 and IL‐1β. It also inhibits activation of the NLRP3/caspase‐1/IL‐1β signalling pathway, thereby reducing inflammatory responses and liver damage.^161^

Alcoholic liver injury is a common type of drug‐induced liver injury. Lactic acid directly causes macrophages to lactate the K33 site of neural precursor cell down‐regulated protein 4 (NEDD4), thereby reducing the interaction between NEDD4 and caspase‐11, which prevents ubiquitination and increases the protein level of caspase‐11, which in turn exacerbates secondary injury of alcoholic liver injury and upregulates atypical pyroapoptosis of macrophages.^162^

Macrophages are recruited and activated, a key link in the inflammatory cytotoxic cycle that leads to widespread cell death in livers that suffer liver ischemia/reperfusion (LI/R) injury. Hepatocyte HSPA 12A serves as a new regulatory factor that reduces inflammatory activation and macrophage chemotaxis by preventing glycolysis mediated lactic acid phosphorylation of HMGB1 and hepatocyte secretion. This effect prevents liver damage caused by liver ischemia/reperfusion LI/R.^163^

#### 5.1.2 Role in lung injury

ALI is a severe inflammatory response that damages the vasculature and alveolar epithelial cells. Curcumin (Cur) and resveratrol (RV) have been used to treat lung injuries by specifically delivering them to inflammatory lungs. By modifying the polarization of M2 macrophages and lowering the amounts of histone lactylation in macrophages caused by LPS, Cur–RV may alleviate ALI.^164^

METTL3 expression can be downregulated or inhibited to reduce sepsis‐related lung damage associated with ALI. Lactate upregulates p300‐mediated H3K18la in the METTL3 promoter region, hence stimulates METTL3 transcription. Furthermore, short‐term lactate stimulation boosts sepsis‐associated lung damage, causes mitochondria‐dependent ferroptosis and activates oxidative and mitochondrial stress mechanisms.^165^

#### 5.1.3 Role in renal injury

In sepsis‐associated acute kidney injury, LPS stimulation has been demonstrated to increase H3K18 and Ezrin lactylation, thereby activating the RhoA‐ROCK1‐Ezrin signalling pathway. This activation subsequently results in increased apoptosis, NF‐κB activation and elevated renal damage indicators. Furthermore, lactate exacerbates Ezrin‐mediated renal injury by promoting lactylation at the Ezrin K263 site.^166^

### 5.2 Role in chronic inflammatory diseases

#### 5.2.1 Role in liver fibrosis

Hepatic stellate cells undergo a trans differentiation process, also known as the activation process. They are the main source of myofibroblasts that release matrix proteins and play a major role in the development of liver fibrosis. These myofibroblasts are derived from liver stellate cells transdifferentiated and can release matrix proteins.^167^ Lactylation of histone H3K18 affects the progression of liver fibrosis by promoting the expression of SOX9, a transcription factor associated with the fibrosis process. This process enhances the migration and growth of hepatic stellate cells and the production of extracellular matrix.^168^

Recent studies have shown that insulin‐like growth factor 2 messenger ribonucleic acid binding protein 2 (IGF2BP2) plays a key role in the development of liver fibrosis. It regulates its activation by reconfiguring the glycolytic metabolism of hepatic stellate cells. The lactic acid produced by glycolysis serves as a substrate for histone lactylation in activated hepatic stellate cells, resulting in increased levels and accumulation of H3K18la and H4K12la labelling in M1 macrophages. Blocking histone lactylation in vivo has been shown to reduce the severity of liver fibrosis.^169^

#### 5.2.2 Role in pulmonary fibrosis

The fibrotic characteristics of lung muscle fibroblasts are mainly triggered by their metabolic transformation, which is significantly marked by increased glycolysis. Experimental evidence demonstrates that p300‐mediated histone lactylation, facilitated by elevated lactate levels, significantly stimulates fibrotic gene expression in pulmonary macrophages, thereby amplifying their profibrotic activity.^170^ Studies have shown that exposure to PM2.5 increases lactic acid levels in macrophages, thereby promoting histone lactic acid modification. This modification enhances the transcription of genes associated with fibrosis. Results pro‐fibrotic factors such as TGF‐β and VEGFA secreted by macrophages can promote EMT of pulmonary epithelial cells, which ultimately leads to the development of pulmonary fibrosis.^171^

Mechanistic investigations further indicate that lactate produced from myofibroblasts employs MCT1 to augment overall Kla and particularly increase H3K18la levels in arsenic‐related idiopathic pulmonary fibrosis. The H3K18 lactylation change expedites fibrotic progression by activating the YTHDF1/m6A/NREP signalling pathway, which facilitates the fibroblast‐to‐myofibroblast transition.^172^

Silicosis, which is characterized by irreversible fibrosis and persistent lung inflammation, can result from long‐term inhalation of ambient crystalline silica dust (CS).^173^ By increasing lactate, CS raises histone lactylation levels, which in turn trigger NLRP3‐dependent macrophage pyroptosis, which in turn causes pulmonary inflammation and fibrosis. Glycolytic inhibitors 2‐Deoxy‐d‐glucose (2‐DG) can reduce pulmonary inflammation and fibrosis and block CS‐induced macrophage pyroptosis.^174^

#### 5.2.3 Role in renal fibrosis

In renal fibrosis, altered PKM2 activity leads to an abrupt increase in lactate concentrations within renal cells. Excessive lactate induces alterations in histone lactylation, notably H3K18la, subsequently activating TGF‐β1 production. TGF‐β1 is a crucial multifunctional cytokine that affects macrophages through paracrine or autocrine mechanisms, subsequently activating the Smad3 signalling pathway. When Smad3 is activated, it promotes the transformation of macrophages from inflammatory M1 to M2, thereby supporting tissue healing and extracellular matrix development. The alteration in polarization facilitates the fibrosis process during prolonged inflammation and stimulation. M2 macrophages produce large quantities of extracellular matrix elements such as collagen and fibronectin, and also inhibit the activity of matrix metalloproteinases. This condition can lead to excessive accumulation of extracellular matrix within the kidney, forming fibrotic scars that interfere with the normal structure and function of the kidney, thereby accelerating the development of fibrosis.^175^

## 6 MODULATION OF MACROPHAGE ACTIVITY IN OTHER DISEASES BY LACTYLATION MODIFICATION

Lactylation, a key PTM, regulates immune function and homeostasis.^72^ This modification further promotes the progression of tumours, inflammatory diseases and related diseases.^176^ Mechanistically, lactylation‐mediated alterations exert regulatory effects on macrophage polarization, thereby influencing disease progression.

### 6.1 Role in non‐alcoholic fatty liver disease

In non‐alcoholic fatty liver disease, the mitochondrial pyruvate carrier (MPC1) suppression alters hepatocyte lactate levels and modifies lactylation patterns of multiple proteins, with fatty acid synthase (FASN) being most prominently affected. The reduction in hepatic lipid accumulation associated with MPC1 downregulation is mediated through lactylation at the FASN K673 residue, which functionally inhibits FASN activity. Intriguingly, although MPC1 deficiency‐induced lactate, concurrent mitochondrial protective mechanisms and a unique M2 macrophage polarization phenotype—characterized by diminished inflammatory cytokine production—collectively mitigate tissue inflammation.^177^

### 6.2 Role in metabolic dysfunction‐associated steatotic liver disease

In metabolic dysfunction‐associated steatotic liver disease, a study by Li et al. showed elevated levels of HK2 and H3K18 lactylation in liver macrophages in both patient and mouse models. HK2 promotes glycolytic activity and lactic acid production, which in turn promotes histone lactic acid, forming a self‐reinforcing cycle involving HK2, glycolysis and H3K18la. This feedback loop exacerbates metabolic imbalances and promotes the transition of macrophages to a pro‐inflammatory M1 state. Targeting deletion of Hk2 or blocking the activity of the transcription factor HIF‐1α in myeloid cells disrupts this cycle, leading to reduced inflammation and reduced activation of M1 macrophages. These findings suggest that targeting this feedback loop could open new therapeutic avenues for metabolic dysfunction‐associated steatotic liver disease.^178^

### 6.3 Role in diabetes‐related conditions

In individuals with type 2 diabetes, aberrant overexpression of MCT4 on cardiomyocyte plasma membranes drives excessive lactate efflux from cardiac cells. Interestingly, this efflux phenomenon is associated with a significant increase in intracellular lactic acid concentration, which leads to enhanced lactylation of histones at the H3K18 and H4K12 positions. Specifically, lactate‐mediated upregulation of H4K12la lactylation promotes HIF‐1α transcription, thereby amplifying macrophage pro‐inflammatory polarization and triggering inflammatory cell infiltration within the cardiac microenvironment. Notably, pharmacological inhibition of MCT4 in type 2 diabetic models significantly attenuates inflammatory macrophage recruitment, improves cardiac functional parameters and reduces both oxidative stress and histopathological myocardial damage.^179^

Lactylation modification enhances the interaction between cyclin dependent kinase 2 and fat mass and obesity‐associated proteins (FTO) in diabetic retinopathy, which may exacerbate microvascular complications. This process is regulated by elevated H3K18la lactylation levels that drive FTO overexpression. To address this pathological cascade, a macrophage membrane‐coated nanoplatform utilizing PLGA‐Di1 has been engineered for systemic delivery of the FTO‐specific inhibitor FB23‐2, demonstrating therapeutic potential for mitigating disease progression.^180^

### 6.4 Role in Alzheimer's disease

In Alzheimer's disease (AD), macrophages in the central nervous system, including resident microglia and border‐associated macrophages located in the meningeal, choroid plexus and perivascular areas, show a transition to pro‐inflammatory microglia activation.^181^

Senescent microglia, together with elevated lactate levels, promotes enhanced lactic acid phosphorylation of H3K18, which facilitates stronger attachment to the promoters of Rela (p65) and NFKB1 (p50), which in turn triggers the NF‐κB signalling pathway. This activation leads to increased expression of aging‐associated secretory phenotypic factors such as IL‐6 and IL‐8, which ultimately contributes to the progression of AD.^182^ Additionally, in AD, the messenger RNA P.G_45033, which is highly expressed by *Porphyromonas gingivalis*, can promote the reorganization of glucose metabolism in macrophages. Transfecting macrophages with this mRNA raises lactate levels, facilitating histone lactylation and augmenting Aβ levels, likely aggravating AD.^183^

### 6.5 Role in myocardial repair

As the heart recovers from a myocardial infarction, lactate transported by MCT1 triggers histone lactylation at the H3K18 site. This epigenetic modification plays a key role in controlling macrophage gene expression, ultimately enhancing their tissue repair capabilities. Following a myocardial infarction, repair genes like Lrg1, Vegf‐a and IL‐10 are activated. Moreover, MCT1‐mediated lactate transfer enhances the dual repair functions of monocytes‐macrophages, particularly their anti‐inflammatory and pro‐angiogenic properties.^184^

### 6.6 Role in myopia

Scleral hypoxia is a critical contributor to the progression of myopia. Hypoxia‐induced enhancement of glycolysis leads to lactate generation, which subsequently culminates in myopia. In myopia, rising lactate levels cause increased H3K18 histone lactylation, activating the Notch1 gene expression. This mechanism promotes the transformation of scleral fibroblasts into muscle fibroblasts, which play a role in the progression of myopia.^185^

### 6.7 Role in secondary hemophagocytic lymph histiocytosis

Secondary hemophagocytic lymphohistiocytosis (sHLH) is an uncommon and serious condition that impacts youngsters. It is marked by macrophage hyperactivation and a cytokine storm. Circulating METTL3 (circMETTL3) levels are heightened in plasma exosomes from sHLH patients, indicating its diagnostic potential. The researchers found that the circular RNA circMETTL3 produces a new peptide called METTL3‐156aa, which binds to LDHA and promotes the polarization of macrophages towards the M1 phenotype by enhancing glycolytic activity in immune cells. Lactate enhances SRSF10 expression through lactylation, hence augmenting circMETTL3 production. The signalling pathway involving the circular RNA circMETTL3, the protein METTL3‐156aa, the lactate dehydrogenase LDHA, lactic acid and the serine/arginine rich splicing factor SRSF10 forms a self‐reinforcing cycle that may become a new potential target for the treatment of sHLH.^186^

## 7 COMPARATIVE ANALYSIS OF LACTYLATION MECHANISMS UNDER THE BACKGROUND OF CANCER AND INFLAMMATION

The regulatory mechanism of lactylation modification in cancer and inflammatory illnesses is predicated on elevated lactate levels. The synthesis of lactic acid arises from hypoxia and elevated glycolytic activity (exemplified by the Warburg effect in tumours or inflammatory glycolysis in immune cells), which supply the fundamental substrate for lactylation. Lactylation modification of histone sites, particularly H3K18, frequently occurs in numerous disorders, including the pro‐tumourigenic M2 polarization phenotypic change in cancer and the regression response during inflammation.^80^, ^90^, ^102^, ^166^, ^168^, ^182^

### 7.1 Mechanism of lactylation in cancer background

Lactylation modification, a significant epigenetic alteration process, facilitates tumourigenesis and progression inside the TME. In colorectal, gastric and ovarian cancers, the lactylation of the histone H3K18 region in tumour‐associated macrophages perpetually facilitates the polarization of TAMs towards an M2‐like phenotype by modulating gene expression programmes. This polarized condition not only diminishes the anti‐tumour immune response but also facilitates tumour cell proliferation, invasion and EMT through the secretion of different proteins.^80^, ^91^, ^187^ Moreover, lactylation modification sustains the elevated lactate levels within the TME via a positive feedback loop, which further amplifies lactate generation by upregulating glycolytic enzyme expression, thereby creating a pro‐tumour vicious cycle.^98^

### 7.2 Mechanism of lactylation in inflammatory background

Conversely, in inflammatory or autoimmune illnesses, lactylation frequently promotes anti‐inflammatory or pro‐regressive phenotypes. H3K18 lactylation in macrophages can enhance the expression of anti‐inflammatory genes including Arg1 and IL‐10, facilitating inflammation resolution and tissue repair.^145^ This indicates that the identical alteration may yield contrasting consequences in various disease contexts: in cancer, it facilitates disease development by suppressing anti‐tumour immunity, whereas in excessive inflammatory conditions, it may mitigate tissue damage and enhance healing. The lactylation modification of non‐histone proteins further underscores the contextual uniqueness of their mode of action. In RA, the lactylation of lysine 62 in pyruvate kinase M2 (PKM2) influences its metabolic function and nuclear translocation, thereby modulating the inflammatory response^138^; in sepsis, the lactylation of HMGB1 protein amplifies the release of inflammatory mediators and intensifies systemic inflammation.^21^

## 8 TARGETING LACTYLATION POSSIBILITY

### 8.1 Potential drug targets for lactate and lactylation in regulation of macrophage polarization in tumours

Lactate influences macrophage functionality in multiple ways within the high lactate microenvironments of tumours. It promotes macrophage polarization by facilitating the uptake of MCTs or serving as a signalling cue to trigger GPR132‐mediated signal transduction. This process shifts macrophages towards the M2 phenotype by suppressing genes associated with the M1 type and enhancing those linked to M2, through various pathways including ERK/STAT3,^188^, ^189^ mTORC1/ATPV0D2/HIF‐2α,^190^ MCT‐HIF1α^191^ and cAMP‐ICER‐NF‐κB.^192^ Augmented M2 macrophage polarization facilitates tumour angiogenesis, correlates with immune evasion and accelerates tumour growth.

Recent research has highlighted lactate, its signalling mechanisms and macrophages as potential new avenues for cancer therapy. In particular, enzymes like LDH and transporters such as MCT are emerging as promising targets in the fight against tumours.^193^ Reagents like AR‐C155858 targeting MCT1 or MCT2 and SR1380 for MCT1, which are typical inhibitors of MCTs, exhibit anticancer properties.^194^ Oxalate, Gossypol, FX11, *N*‐hydroxypiperidine (NHI) and quinazoline 3‐sulfonamide are among the most common LDH inhibitors. In cancer treatment, these drugs suppress LDHA expression, thereby inhibiting cell proliferation, migration and tumour formation.^195^

Lactate‐derived lactylation modification regulates macrophage polarization. Elevated lactate levels in tumours lead to increased lactylation, which correlates with poor outcomes in cancer patients.^83^ Lactate‐induced H3K18 lactylation facilitates M2 macrophage polarization and expedites tumour progression.^80^, ^90^ In cancer treatment, blocking H3K18la has been shown to restore sensitivity to cisplatin, suggesting a potential new method for bladder cancer therapy.^125^ In CRC treatment, inhibiting H3K18 lactylation may enhance bevacizumab's clinical efficacy. A critical molecular axis involving SRSF10, MYB, glycolysis and H3K18la underpins immune evasion and anti‐PD‐1 treatment resistance in liver cancer. Knocking down SRSF10 may shift macrophage polarization away from the M2 phenotype, boosting the effectiveness of PD‐1 inhibitors in hepatocytes.^104^

Researchers can formulate immunotherapeutic agents for cancer treatment by focusing on lactate metabolism, intervening in lactylation modifications, examining genes that govern the lactylation process, adjusting lactate and lactylation levels and modifying the polarization state of macrophages within TME via lactate regulation, signalling pathways and lactylation modification mechanisms. It is anticipated that the implementation of these strategies will lead to the development of more effective methods for the treatment of cancer, particularly in enhancing chemotherapy and the effectiveness of immunotherapy, indicating significant potential.

### 8.2 Potential drug targets for lactate and lactylation in controlling macrophage polarization in inflammation

In the context of inflammation, lactate affects the phenotypic and functional transition of macrophages via multiple signalling mechanisms. Lactate activates the AMPK/LATS/YAP^196^ and Wnt/β‐catenin^197^ pathways, which inhibit NF‐κB in a direct or indirectly, hence exerting pro‐inflammatory effects. The dynamic relationship between lactate levels and macrophage polarization plays a pivotal role in both triggering and advancing inflammatory diseases,^198^ presenting promising opportunities for therapeutic intervention.^199^

**FIGURE 4:**
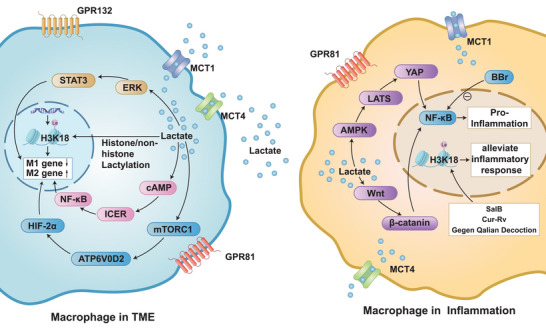
The lactate signalling process and the role of lactylation in macrophages amidst inflammation and tumourigenesis. Researchers have identified three primary lactate‐dependent signalling pathways in macrophages within the tumour microenvironment: the mTORC1/ATP6V0D2/HIF‐2α axis, the ERK/STAT3 pathway and the cAMP/ICER/NF‐κB cascade. Moreover, lactylation—whether on histone proteins or other cellular constituents—primarily targets the H3K18 locus, producing significant biological effects. Two principal signalling pathways activated by lactate during inflammatory responses have been identified: the AMPK/LATS/YAP/NF‐κB pathway and the Wnt/β‐catenin pathway. Both routes generally enhance inflammatory activity; however, their effects can be alleviated by berberine (BBR). Furthermore, substances including salvianolic acid B (Sal B), a combination of curcumin and resveratrol (Cur–RV), and Gegen Qianqian Decoction can inhibit H3K18 lactylation, thus attenuating inflammatory reactions.

Lactate‐derived lactylation significantly influences macrophage polarization in inflammatory conditions, and targeting this mechanism presents a distinctive treatment strategy. Recent studies have illuminated this trajectory. For instance, Cur–RV may regulate M2 macrophage polarization by reducing histone lactylation,^164^ and Gegen Qinlian Decoction can modulate macrophage polarization and lactylation levels to mitigate inflammatory responses.^154^ Sal B can decrease histone H3K18la in M1 macrophages at the levels of LDHA and inflammation‐related genes (Figure 4).^161^ These results suggest that lactate and lactylation modulation could influence macrophage polarization, offering therapeutic potential for inflammatory diseases.

### 8.3 Discussion and future prospects

Future research could focus on developing immunotherapeutic drugs or targeted therapies that target specific lactylation sites, or using lactylation scores to predict immune evasion, cancer progression and prognosis. The exact mechanisms by which lactylation modification regulates macrophage polarization and the specific lactylation sites involved are still unclear. A ground‐breaking study in cell has revealed the pivotal role of lactylation in immune system regulation. When analysing the immune response in BCG‐vaccinated subjects, researchers found that lactylation boosts H3K18la levels, steering macrophages towards the M2 phenotype. This shift not only dampens inflammation but also promotes an anti‐inflammatory state. Both lab experiments and animal trials confirm that lactylation dramatically influences macrophage behaviour, opening doors for cutting‐edge immunotherapy approaches. Scientists are now digging deeper into how lactylation drives the switch from pro‐inflammatory M1 to anti‐inflammatory M2 macrophages—with early evidence pointing to the protein TRIM33 as a potential key player in this transition. In oncology, a correlation exists between poor tumour prognosis and elevated lactylation levels, but the degree of this correlation is uncertain, as is the potential impact of low lactylation levels on carcinogenesis.

Moreover, questions remain about the specificity of H3K18 lactylation in macrophage polarization and the lactate concentration required to trigger lactylation. Our research findings suggest that modifications in lactylation significantly contribute to treatment resistance in ovarian cancer. Subpopulations of drug‐resistant cancer cells have increased lactic acid build‐up, oxidative phosphorylation and glycolytic activity. Lactylation, particularly H3K18 lactylation, facilitates M2 polarization and enhances macrophage production of CCL18. As a result, it boosts ovarian cancer cell growth and movement. S100A4 and ALDH1A1 are two genes prevalent in drug‐resistant cells. They are associated with immunological and metabolic activities. To truly unpack the intricacies of lactylation's positive impact on the modification process, and how it intertwines with other epigenetic markers such as m6A during macrophage differentiation, a more comprehensive investigation is in order.

In conclusion, numerous opportunities exist for studying how lactylation modifications affect macrophage regulation. The mechanisms of lactylation modification need further study, and their importance in macrophage polarization should be continuously emphasized. This research may yield innovative immunotherapeutic and personalized treatment approaches, enhancing human health and disease management.

## 9 CONCLUSIONS

Macrophages are crucial effector cells in the immune system. They maintain tissue homeostasis and respond to immunological stressors. Macrophages exhibit significant plasticity in their functions. There are two main types: M1 macrophages that promote inflammation and M2 macrophages that counteract it. M1 macrophages assist in combating infections, initiate inflammatory responses, induce tissue damage and inhibit cancer proliferation. M2 macrophages, conversely, excel in reducing inflammation, facilitating tissue repair, combating infections and regulating immune responses tumour angiogenesis is promoted, facilitating tumour progression. The preservation of immune homeostasis depends on the dynamic equilibrium between M1 and M2 macrophages. Disruption of this balance under pathological conditions, whether favouring persistent inflammation (M1 dominant) or excessive repair/immune suppression (M2 dominant), serves as a fundamental impetus for the emergence and progression of various diseases. Consequently, targeting and modulating macrophage polarization to encourage differentiation towards pathogen clearance or disease progression inhibition has become a pivotal strategy for addressing inflammation‐related diseases and malignant tumours.

In this regard, lactylation modification, a novel addition to the epigenetic regulatory repertoire, is rapidly gaining prominence in immune metabolism research. This modification process is governed by specific “writers,” “readers” and “erasers.” This post‐translational modification independently regulates gene expression and interacts with other modifications like phosphorylation and acetylation. Together, they affect protein structure, function and activity, thereby influencing gene expression and disease development. Lactate supercharges the activation of M2 macrophage's homeostatic genes during the latter part of their M1 polarization by promoting histone lactylation at the H3K18 spot. Elevated lactate levels in the TME are taken up by TAMs via MCT1, which depends on H3K18 lactylation to directly induce the expression of M2 functional genes, thus promoting a pro‐tumour and immunosuppressive phenotype in TAMs. This mechanism has been confirmed in multiple cancer types. In the central nervous system, microglial responses to lactate signalling resemble those of TAMs: lactate promotes M2 polarization and facilitates tumour recruitment. However, in certain models of CNS inflammation, lactate can also induce a pro‐inflammatory phenotype in microglia by upregulating pro‐inflammatory factors like IL‐23. This indicates that lactate regulation may exhibit microenvironment‐dependent bidirectional plasticity. Liver transplantation models demonstrate that M2 polarization of Kupffer cells is essential for promoting immune tolerance and minimizing acute rejection. The study did not directly identify lactylation; however, it established that ANGPTL4 from liver cells inhibits NF‐κB signalling via paracrine pathways, leading to the M2 phenotype transition in Kupffer cells. The findings indicate that lactylation modification, in conjunction with other microenvironmental signals, influences macrophage polarization. Lactylation plays a critical role in the regulation of macrophage phenotype and is significantly associated with tumour progression as well as the onset and progression of inflammatory diseases. An in‐depth analysis of the macrophage polarization network mediated by lactylation will establish a theoretical foundation for the development of targeted therapeutic strategies focused on immune cell reprogramming.

Although lactylation modification research is currently moving quickly forward, more work is still needed to identify the precise alteration sites and underlying mechanisms. It is anticipated that the creation of novel markers will offer fresh approaches to clinical targeted lactylation modification therapy, improving the management of disease on a larger scale. Scientists are trying to identify the signalling mechanisms that control gene expression and find lactylation modification sites in many cell and tissue types. Moreover, we must examine the intricate interplay between lactylation modification and other alterations, as well as its impact on the phenotypic metamorphosis of macrophages. We must determine how lactylation influences disease development and assess the effectiveness of targeting key lactate enzymes as a therapy in animal studies.

We must acknowledge that certain obstacles persist in the advancement of this research. Focussing solely on lactylation changes may be insufficient to clarify all cases or provide universal therapeutic benefits due to the complexity of macrophage phenotypes and the heterogeneity of the cancer immune microenvironment. Macrophages frequently display a continuous spectrum instead of a straightforward M1/M2 dichotomy. The efficacy of single‐target methods is markedly compromised by substantial discrepancies in immune microenvironment attributes—such as immunological profiles, metabolic features and signalling pathways—across different malignancies and within patients of the same tumour subtype.

Therefore, future research must broaden its scope. Lactylation modification should be examined within the broader framework of macrophage metabolic reprogramming, particularly its interplay with key metabolic pathways such as HIF‐1 α. Additionally, the interaction of lactylation with other epigenetic modifications, including acetylation and methylation, as well as critical transcription factors and cytokine signalling networks, warrants investigation to elucidate the functional status of macrophages. Furthermore, it is essential to consider how signals and metabolites from tumour cells or other immune cells, beyond lactate alone, influence lactate levels and macrophage activity. In conclusion, gaining a comprehensive understanding of how lactylation influences macrophage polarization and their ability to adapt is essential for unravelling the underlying factors of various diseases and developing targeted interventions.

## AUTHOR CONTRIBUTIONS

Houhua Guo and Liancheng Zhu wrote the main manuscript text. Houhua Guo, Jianlei Bi and Nannan Luan prepared Figures 1 and 2. Jian Gao and Xiaoao Pang prepared Figures 3 and 4. All authors reviewed and approved the final manuscript.

## CONFLICT OF INTEREST STATEMENT

The authors declare no conflicts of interest.

## ETHICS STATEMENT

Ethical approval is not applicable to this study.
